# The History of Surgical Stabilization of Rib Fractures (SSRF)

**DOI:** 10.1016/j.sipas.2022.100084

**Published:** 2022-04-20

**Authors:** Youssef Shaban, Madelyn Frank, Sebastian Schubl, Claire Sakae, Anushka Bagga, Mennatalla Hegazi, Ronald Gross, Andrew Doben, Jeffry Nahmias

**Affiliations:** a University of California, Division of Trauma, Burns & Surgical Critical Care, 333 City Blvd West, Suite 1600, Irvine, Orange, CA, 92868, United States; b University of California, School of Medicine, Irvine, United States; c Trinity Health of New England, Saint Francis Hospital and Medical Center, Division of Acute Care Surgery, Hartford, CT 06105, United States

**Keywords:** History of surgical stabilization of rib fractures (SSRF), Rib fixation, Flail chest, Rib plating, Rib fractures, Open reduction and internal fixation (ORIF) of rib fractures

## Abstract

Responsible for approximately 35% of all trauma-related deaths in the United States, thoracic trauma is one of the leading causes of death among trauma patients. Furthermore, traumatic rib fractures represent the most frequently encountered injury following thoracic trauma with mortality rates ranging from 8% among the elderly to 13% for patients with a flail chest. This manuscript reviews the history of SSRF as well as the contributions of the pioneering surgeons who championed this treatment. Rib fractures are a marker for severe injury as indicated by a recent National Trauma Data Bank (NTDB) retrospective analysis of 564,798 patients with one or more rib fractures. Approximately half of these patients were found to have multiple injuries with worse outcomes observed in patients with polytrauma and flail chest [1]. In addition, age, male gender, injury severity score (ISS), Glasgow Coma Scale (GCS), preexistent comorbidities, and number of rib fractures are independently associated with significantly higher rates of morbidity and mortality [1, 2]. Recently, studies have demonstrated surgical stabilization of rib fractures (SSRF) improves outcomes for ventilated as well as non-ventilated patients with flail chest, elderly patients, and select patients with multiple rib fractures without a flail injury or non-flail fracture pattern [2–6]. SSRF applies orthopedic principles of reduction and fixation to restore the architecture of the thoracic skeleton and re-establish normal respiratory physiology and minimize pain [7]. There has been a recent increase in prevalence of SSRF operations, however, SSRF is not a new technique, and progress has been anything but mundane or linear [3, 6]. This manuscript reviews the history of SSRF (Figure 1) as well as the contributions of the pioneering surgeons who championed this treatment. This review was conducted utilizing multiple national experts and a thorough literature review of related SSRF was performed. The sources chosen are considered by the authors to be highly influential and include the first publication for each distinctive method of SSRF.

## Highlights

• The earliest account of open reduction and internal stabilization of rib fractures comes from Dr. Charles Locke Scudder in 1900 when he published, *the treatment of fractures*.
• Surgical stabilization of rib fractures (SSRF) has evolved over 120 years with numerous advancements and lessons learned along the way.
• Materials have evolved from sutures to wires to external and internal plates.
• Rib fixation methods have evolved from open suture and wire cerclage to minimally invasive video assisted thoracoscopic surgery internal plating.

## Inrtroduction

Responsible for approximately 35% of all trauma-related deaths in the United States, thoracic trauma is one of the leading causes of death among trauma patients. Furthermore, traumatic rib fractures represent the most frequently encountered injury following thoracic trauma with mortality rates ranging from 8% among the elderly to 13% for patients with a flail chest [1].

Rib fractures are a marker for severe injury as indicated by a recent National Trauma Data Bank (NTDB) retrospective analysis of 564,798 patients with one or more rib fractures. Approximately half of these patients were found to have multiple injuries with worse outcomes observed in patients with polytrauma and flail chest [1]. In addition, age, male gender, injury severity score (ISS), Glasgow Coma Scale (GCS), pre-existent comorbidities, and number of rib fractures are independently associated with significantly higher rates of morbidity and mortality [1,2].

Recently, studies have demonstrated surgical stabilization of rib fractures (SSRF) improves outcomes for ventilated as well as non-ventilated patients with flail chest, elderly patients, and select patients with multiple rib fractures without a flail injury or non-flail fracture pattern [2,3,4,5,6].

**Fig. 1:**
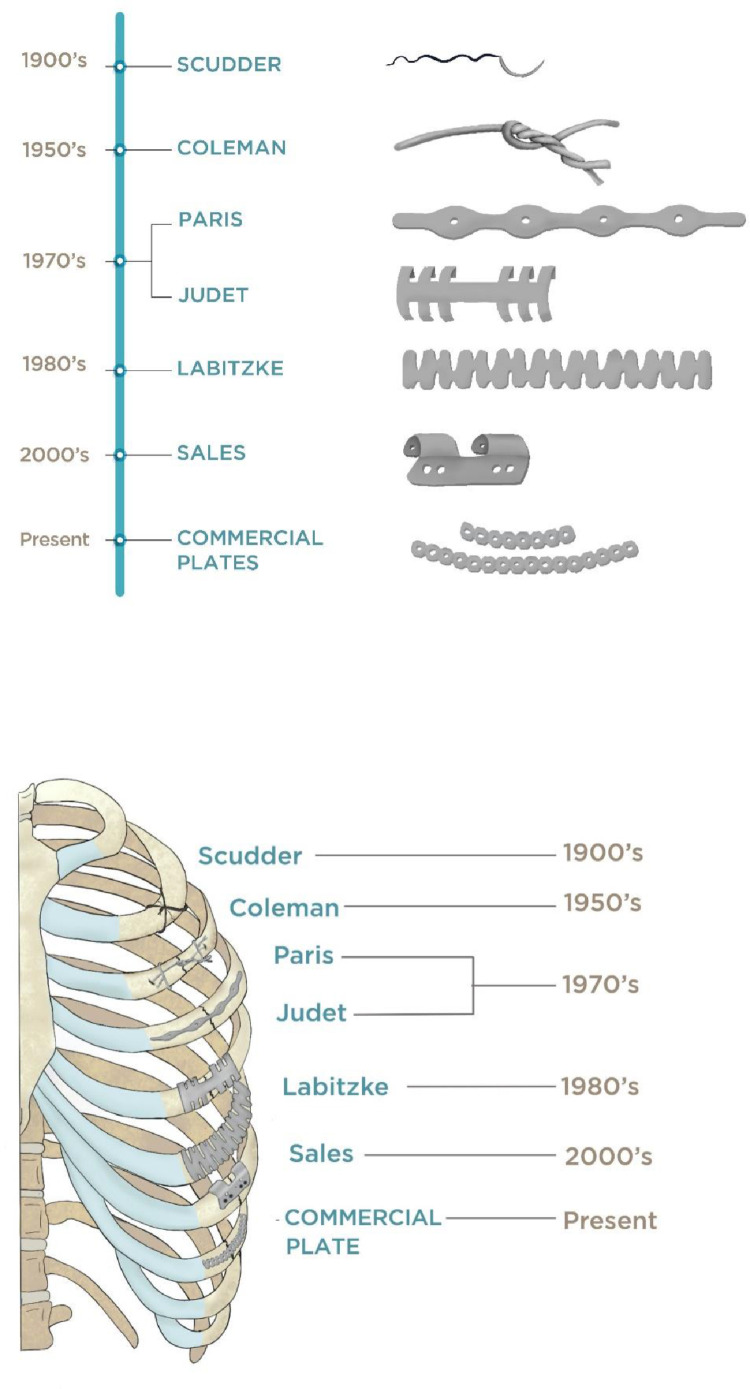
Timeline illustrations depicting the evolution of Surgical Stabilization of Rib Fractures (SSRF) techniques. The 1900 era depicts the first reported SSRF utilizing suture cerclage by Dr. Charles Locke Scudder. The 1950′s portrays wire cerclage demonstrated by Coleman and Coleman. 1970′s depicts the first percutaneously inserted self-made stainless-steel plate (one of the earliest accounts of a minimally invasive approach) followed by the Judet plate which utilized a clamping mechanism via struts or flat hooks that grasp the rib superiorly and inferiorly. The 1980′s illustrates Labitzke's novel titanium-based clamping plate. In the early 2000′s Sales et al. manufactured the U plate. Present day illustrates commercially available low-profile titanium plates that achieve rib fixation via bi-cortical screws.

SSRF applies orthopedic principles of reduction and fixation to restore the architecture of the thoracic skeleton and re-establish normal respiratory physiology and minimize pain [7]. There has been a recent increase in prevalence of SSRF operations, however, SSRF is not a new technique, and progress has been anything but mundane or linear [3,6]. This manuscript reviews the history of SSRF (Fig. 1) as well as the contributions of the pioneering surgeons who championed this treatment.

This review was conducted utilizing multiple national experts and a thorough literature review of related SSRF was performed. The sources chosen are considered by the authors to be highly influential and include the first publication for each distinctive method of SSRF.

## Timeline

### 1900–1950s: Suture and wire cerclage

The earliest account of open reduction and internal stabilization of rib fractures comes from the Massachusetts born Dr. Charles Locke Scudder, the namesake for the Scudder Oration on Trauma. Dr. Scudder also helped develop the American College of Surgeons (ACS) Committee on Treatment of Fractures, which has grown into the modern-day ACS Committee on Trauma (COT). In 1900, he published, *The Treatment of Fractures,* a detailed text regarding the management of fractures of the face, spine, pelvis, extremities, and chest wall. In this, Scudder recommended open incision and immobilization with suture for comminuted or greatly displaced rib fractures [8].

However, it was not until the halfway point of the 20th century at the Medical College of Virginia, that Coleman et al. reported a more durable method of treatment for 15 patients with flail chest injuries, performing open reduction and wire fixation in two planes [7,9].

### 1960–1970s: Introduction of plate fixation

The 1960s brought upon the first account of using a plate and intramedullary wires to stabilize the chest. Silar recorded 5 cases with classic anterior flail injuries with paradoxical movement which were controlled with the application of a sternal plate and intramedullary placed Kirschner wires [7].

Historically, the main approach to perform SSRF was a traditional thoracotomy with complete division of the latissimus dorsi muscle. These large incisions were quite morbid leading many in the surgical community to question the benefit of SSRF [6]. In 1972, one of the earliest accounts of minimally invasive SSRF took place in Valencia, Spain by Paris et al. who utilized percutaneous inserting self-made stainless-steel plates. This was performed via two small incisions in a subpectoral plane and fixating the plates by wire cerclage [10].

Soon after in 1973 a novel SSRF technique was introduced in France. Judet's technique fixated the reduced ribs using anteriorly situated metal plates held in place using a clamping mechanism via struts or flat hooks that grasp the rib superiorly and inferiorly. This novel technique helped avoid the potential risk of iatrogenic lung laceration while placing screws.  However, critics emphasize that the inferior struts as well as cerclage wire impinge on the intercostal neurovascular bundle, causing nerve injury and chronic pain [5,11].

### 1980s Titanium plates

A German surgical trailblazer named Labitzke created the first known titanium-based clamping plate in 1980, with the ability to contour to the unique rib shape [12]. Prior to titanium-based plates, surgeons used a wide variety of osteosynthesis plates including stainless steel, which was exceedingly rigid and frequently caused postoperative chest wall pain or loosening of screws, thereby necessitating removal [10].

### 2000s-Future: New methods and expanding indications

In 2008 Sales et al. developed the U plate now called *RibLoc*™ by combining an anterior plating system which is secured via cortically placed screws and the novel Judet strut grasping mechanism [5].

In addition, during the early 21st century a wide proliferation of commercial SSRF options were developed with the help of many surgeons. These systems, however, share an essential fundamental property of utilizing low-profile titanium-based plates that easily contour to a specific rib while also providing semi-rigid fixation. Examples of the most widely used systems include *MatrixRib*™, *L1 Rib system*™, *StraCos*™ system, *RibFixBlu*™, *NiTi Fixing Plates*™, and *Ribfix Advantage*™. Only one of the commercial systems has FDA approval for intrathoracic use.

In addition to novel mechanisms of fixation, reports on use of absorbable plates have begun to emerge. Touted benefits of absorbable plates include the in vivo degradation thereby leaving the patient without any permanent foreign body. However, reports of hardware failures particularly with posteriorly displaced fractures have also surfaced, thus leaving an unclear future for absorbable plates at this time [5,13].

Coupled to increasing technology, there has been a recent push for improved surgical techniques for SSRF and other treatment of rib fractures.  This includes muscle sparing incisions, cryoablation and thoracoscopic-assisted SSRF. Pieracci et al. were the first to report a totally thoracoscopic, intra-pleural SSRF [6]. This was followed in 2018 by Merchant and Onugha who described a minimally invasive extra-thoracic video-assisted thoracoscopic SSRF method using balloon dilation to create an accessible extra-thoracic working space [4].

While SSRF has come a long way since the days of Scudder, there remains many areas of needed focus including optimal indications and minimally invasive approaches (i.e., robotic assisted or percutaneous). However, continued innovation appears inevitable based on the trajectory of the past 120 years and rapid evolution over the past two decades.

One major reason has been a significant increase in research.  Tanaka's 2002 publication was a landmark paper [14], but Long and Trunkey (among others) had proposed SSRF in differing forms and for different indications for well over 50 years [15,16,17].  A potential recent catalyst for the adoption of SSRF has been the development of the Chest Wall Injury Society (CWIS).  This multidisciplinary group and its annual scientific assembly have helped foster increased publications and collaborations regarding chest wall injuries, including the publication of the NON-FLAIL trial [3,18,19].

In terms of the future, while the technology of available rib fixation systems has improved vastly, one important future development will be a more individualized approach to SSRF. Utilizing three-dimensional printing and novel plate materials (i.e., carbon fiber, bio resorbable products etc.) we may be able to map exact rib parameters and manufacture fixation plates that are truly individualized to each patient, similar to endovascular devices for aortic surgery [20,21]. Finally, with continued research and improved technology the authors believe risk prediction will eventually become individualized as well, likely with the help of artificial intelligence risk modeling. Overall, the future of rib fracture care appears bright with significant potential for further discovery and improvements in clinical care.

## Declaration of Competing Interest

The authors declare the following financial interests/personal relationships which may be considered as potential competing interests:

Sebastian D. Schubl reports a relationship with Zimmer Biomet that includes: consulting or advisory.

Andrew R Doben has a patent with royalties paid to Zimmer Biomet. Andrew R Doben receives royalties for a product unrelated to this work, however the patent is held by a producer of hardware related to this article.
